# Deficits of Visual Cortex Function in Acute Acquired Concomitant Esotropia Patients

**DOI:** 10.1167/iovs.64.13.46

**Published:** 2023-10-30

**Authors:** Yan Hu, Shenjiang Wang, Lianqun Wu, Sida Xi, Wen Wen, Chen Zhao

**Affiliations:** 1 Department of Ophthalmology and Vision Science, Eye and ENT Hospital, Shanghai Medical School, Fudan University, Shanghai, China; 2 NHC Key Laboratory of Myopia, Fudan University, Shanghai, China; 3 Shanghai Key Laboratory of Visual Impairment and Restoration, Fudan University, Shanghai, China; 4 Department of Radiology, Eye and ENT Hospital, Shanghai Medical School, Fudan University, Shanghai, China

**Keywords:** acquired concomitant esotropia (AACE), functional magnetic resonance imaging (fMRI), amplitudes of low-frequency fluctuation (ALFF), primary visual cortex, dorsal pathway

## Abstract

**Purpose:**

The purpose of this study was to explore the cortical deficits of patients with acquired concomitant esotropia (AACE) using the resting-state functional magnetic resonance imaging (rs-fMRI) technique.

**Methods:**

Rs-fMRI signals from 25 patients with AACE and 25 matched controls were collected. The repeated-measures analysis of variance (RM-ANOVA) test and two-sample *t*-test were used to investigate statistical differences of the amplitudes of low-frequency fluctuation (ALFF) signals and correlation analysis was performed to validate the relationship of signal change and clinical features.

**Results:**

The AACE group showed decreased ALFF in both hemispheres symmetrically (*t* = 0.38, *P* = 0.71), with peak t in both middle occipital gyrus. The ALFF signal from the upper left inferior frontal gyrus was negatively correlated with the age of onset (*r* = 0.62, *P* = 0.0008), and the ALFF signal from the right superior temporal gyrus was negatively correlated with the near work hours (*r* = 0.63, *P* = 0.0008). The ALFF signal in the left fusiform gyrus was positively correlated with both near (*r* = 0.48, *P* = 0.01) and far (*r* = 0.44, *P* = 0.03) deviation, whereas it was only positively correlated with far deviation (*r* = 0.44, *P* = 0.03) in the right. Besides, the age of onset and the near work hour were independent factors of signal changes.

**Conclusions:**

Using the ALFF signal of rs-fMRI, we found functional deficits in the primary visual cortex and dorsal pathway in patients with AACE. There were functional changes in the fusiform gyrus, and the greater the deviation angle, the higher the changing level. These findings reveal the association of AACE and the visual center, giving us more clues about the treatment of AACE.

Acute acquired concomitant esotropia (AACE) is an unusual type of esotropia which occurs in older children and adults. It is characterized by acute onset of esotropia with diplopia,^1^ normal eye movement, and equal deviation in each direction. The golden guideline of treatment is to correct the squint by surgery in order to restore the binocular vision. Most patients with AACE reported immediate satisfaction postoperatively, with diplopia disappeared and eye position corrected.^2^ However, it was reported that 39% of patients with AACE could not restore normal stereopsis after the deviation was corrected.^3^^,^^4^ Furthermore, in clinical practice, there are still 20% of patients who complain of relapse or recurrence.^5^^,^^6^ Besides, in clinical practice and previous clinical research, we also found narrowing of the binocular fusion range and interocular suppression in postoperative patients with AACE.^7^ These phenomena suggested that the pathogenesis of AACE may not only be confined to ocular abnormalities. However, the mechanism of AACE has never been clarified.

Previous animal studies as well as functional magnetic resonance imaging (fMRI) studies in vivo have proposed that abnormal cortical regulations have played an important role in the pathogenesis of non-AACE strabismus. For example, in the animal model of concomitant esotropia, it was found that the number of neural cells regulating stereopsis was reduced, and the metabolic level of the ocular dominant cells (ODCs) decreased significantly.^8^^–^^10^ In a study of the childhood primate, researchers found that if the binocular fusion was disrupted, the primary visual cortex would be damaged and esotropia could be induced then.^11^ Via noninvasive imaging, abnormal changes in the higher visual cortex were also found in patients with concomitant exotropia, such as the volume decrease of the right middle occipital gyrus and the right supramarginal gyrus, which were thought to be related to stereopsis.^12^^–^^14^ The fMRI studies in patients with concomitant exotropia have also found abnormalities in the parietal-occipital sulcus (POS) area, which is involved in both the dorsal and ventral streams and was thought to play an important role in processing object motion and spatial depth information, thus taking an important part in spatial orientation recognition.^15^ The above findings are all about non-AACE strabismus, but the role of cortical regulation in AACE has never been investigated. Some investigators proposed that the long-term near work, especially using smartphones, was associated with the onset of AACE. The excessive near work may lead to abnormalities of the accommodation and vergence function, which were thought to be modulated by the visual cortex.^16^ With the increasing incidence of AACE, especially in Asia,^6^ it is essential to explore the role of cortical function in the pathogenesis of AACE, which will promote our understanding of this disease.

In this study, resting-state fMRI (rs-fMRI) was used to investigate the cortical function abnormality of patients with AACE. Amplitude of low-frequency fluctuation (ALFF) of rs-fMRI is widely applied as a regional indicator for detecting low frequency oscillation (LFO) activities, which were thought to be the indicator of the brain status at resting state. The relationship between ALFF and clinical features was explored by correlation analysis and multivariable linear regression to achieve a further understanding of the pathogenesis of AACE.

## Methods

### Participants

Twenty-five patients with AACE receiving surgery in EENT Hospital Affiliated with Fudan University during March 2021 and December 2021 were enrolled in our study. Inclusion criteria were: (1) diagnosed with AACE, with esodeviation angles between 10 and 80 prism degrees (PD); (2) aged between 16 and 45 years old; (3) best-corrected visual acuity (BCVA) better than 0.1 logMAR; and (4) no history of strabismus surgery. Exclusion criteria were: (1) cerebral tumors; (2) amblyopia; (3) anisometropia greater than 2.50 diopters (D); (4) incomitant strabismus; (5) other ocular diseases except for strabismus and refractive errors; (6) mental, neurologic, or other severe systematic diseases; and (7) not applicable for magnetic resonance examinations, such as having metal implants and claustrophobia.

The inclusion criteria for control subjects: (1) well aligned eye position binocularly; (2) no recessive strabismus; (3) aged between 16 and 45 years old; (4) BCVA better than 0.1 logMAR; and (5) no history of strabismus surgery. Exclusion criteria were: (1) cerebral tumors; (2) amblyopia; (3) anisometropia greater than 2.50 D; (4) diagnosed with strabismus; (5) other ocular diseases except for strabismus and refractive errors; (6) mental, neurologic, or other severe systematic diseases; and (7) not applicable for magnetic resonance examinations, such as having metal implants and claustrophobia.

### Ethics

The study followed the tenets of the Declaration of Helsinki and was approved by the Ethics Review Board of the Eye & ENT Hospital, Fudan University (reference number: 20180618). Informed consent was obtained from all participants or their guardians, when applicable.

### Procedure

#### Ophthalmic Examination

All participants underwent a complete ophthalmic examination, including slit-lamp, cycloplegic refraction, BCVA, fundus examination, eso-deviation angle, and ocular motility. The cerebral tumors were excluded through MRI examination of the head. The degree of eso-deviation was measured by a prism and cover test (PACT) both at far (6 m) and at near (33 cm), with refractive lenses.

#### Eye-Use Information Collection

Online questionnaires were used to collect eye–use information. All subjects were required to fill in the average hours they focused on matters 40 cm in front of their eyes (i.e. smartphone, computer, books, and other relatively static things), per day, before the esotropia onset. Especially, for those who have the demand of vision correction, two kinds of near work hours are collected: one is total near work hours, no matter with or without visual acuity corrected, and the other is near work hours without visual acuity corrected.

#### MRI Acquisition

MRI data were acquired using a 3T Siemens Verio scanner (https://www.siemens-healthineers.com/en-us/magnetic-resonance-imaging/3t-mri-scanner/magnetom-verio) with a 32-channel head coil. Rs-fMRI data were acquired with echo-planar imaging (EPI) pulse sequence (repetition time/echo time [TR/TE], 2000 ms/30 ms; 33 slices; voxel size = 3.5 mm isotropic; 240 volumes]. During scanning, participants were instructed to lie still with their eyes closed and keep resting without any thinking. Structural MRI data were collected using a high-resolution T1-weighted magnetization prepared rapid gradient echo (MPRAGE) sequence (TR/TE, 2200 ms/2.45 ms; voxel size = 1 mm isotropic; 192 slices).

### Data Processing

#### Pre-Processing

Functional images were processed using the SPM12 (Statistical Parametric Mapping 12) toolbox (https://www.fil.ion.ucl.ac.uk/spm/software/spm12) which was supported on the MATLAB 2020a platform (https://ww2.mathworks.cn/products/matlab.html).

Using FSL software (https://fsl.fmrib.ox.ac.uk/fsl/fslwiki) to remove skull and neck tissues. The functional data were preprocessed as follows: discarding the initial 10 volumes for signal equilibrium, slice timing correction, skull removal, head motion correction, and spatial normalization. Images were normalized to the standard Montreal Neurological Institute (MNI) space using a sample-specific template. To generate a sample-specific template, structural T1-weighted images were spatially normalized using the DARTEL^17^ registration, followed by a visual quality check. Linear regression was applied to remove nuisance covariates including the Friston 24^18^ head motion parameters, the white matter signal, global mean signal, and cerebrospinal fluid signal. After spatial smoothing with a 6 mm full-width-at-half-maximum Gaussian kernel, the data were linearly de-trended. Participants with head motion >2 mm of displacements or >2 degrees of rotation around the x/y/z axis would be excluded from further analysis. Mean frame wise displacement, as described by Power, was derived from the re-alignment estimates to evaluate head motion. No significant differences were found between patients with AACE and healthy controls in terms of head motion (Table 1).

**Table 1.** Clinical Characteristics of the AACE Group and the Control Group

	AACE		Control		
	Mean ± SD	Range	Mean ± SD	Range	Statistics
Sex (M: F)	12:13		12:13		χ^2^(1) = 0, *P* > 0.99
Age, y	26.44 ± 7.26	16–42	26.32 ± 3.09	22–36	t(48) = 0.08, *P* = 0.94
Onset age, y	22.7 ± 7.6	12–41	—	—	—
Disease duration, y	3.9 ± 3.9	0.2–14	—	—	—
Near work hours (h/day)	10.2 ± 2.4	6.0–15.0	9.2 ± 2.5	6.0–14.0	U = 229, *P* = 0.09
Uncorrected near work hours (h/day)	2.0 ± 1.0	0.2–3.2	1.4 ± 0.4	0.5–2.0	t(35) = 2.24, *P* = 0.03
Max diopter (D)	−4.41 ± 1.90	−7.50 to +0.00	−3.38 ± 1.64	−6.00 to –0.25	t(48) = 2.06, P=0.04
Min diopter (D)	−3.60 ± 1.80	−6.50 to 0.00	−2.93 ± 1.66	−6.00 to 0.00	t(48) = 1.37, *P* = 0.18
BCVA OD, (log MAR)	0.01 ± 0.02	0.00 to +0.10	0.00 ± 0.03	−0.08 to +0.05	U = 257, *P* = 0.18
BCVA OS, (log MAR)	0.01 ± 0.03	−0.08 to +0.10	0.00 ± 0.03	−0.08 to +0.05	U = 287, *P* = 0.46
Deviation (near, PD)^*^	29.02 ± 15.20	10–78	—	—	—
Deviation (far, PD)^*^	29.60 ± 14.80	10–72	—	—	—

* PD in stream prism.

#### The Amplitude of Low-Frequency Fluctuations Analysis

To investigate frequency-specific ALFF alterations, the entire frequency range (0–0.25 hertz [Hz]) was subdivided into five bands^19^: slow-6 (0–0.01 Hz), slow-5 (0.01–0.027 Hz), slow-4 (0.027–0.073 Hz), slow-3 (0.073–0.167 Hz), and slow-2 (0.167–0.25 Hz). Buzsáki and colleagues classified the oscillatory waves (0.02 to 60 Hz) into 10 frequency bands and termed them “oscillation classes.” Based on their work, Martino et al. and Zuo et al. extended the oscillation classes down to slow-6 to provide a more comprehensive examination of the power spectrum of the intrinsic LFOs. For each voxel, blood oxygen level-dependent (BOLD) time series were transformed to a frequency domain using a fast Fourier transform. The square root of the power spectrum was computed and averaged across each frequency band, which was taken as the ALFF value.^20^ The ALFF value of each voxel was divided by the mean whole-brain ALFF value for each participant to reduce the global effects of variability across individuals. ALFF values were calculated for the typical frequency band (0.01–0.08 Hz) as well as narrowly defined frequency bands.

### Statistical Analyses

#### Primary Analyses

Statistical analyses of demographic and clinical variables were performed on Prism 9 Graphpad software (https://www.graphpad.com/scientific-software/prism/). Kolmogorov-Smirnov test was performed first to check normality. If the parameter passed the normality test, the two-sample *t*-test would be performed, otherwise, the Mann-Whitney test would be performed. Significance was assumed at *P* < 0.05. For comparison of ALFF in the typical frequency band, a two-sample *t*-test was performed. We also did RM-ANOVA for left and right ALFF signals of both groups and paired *t*-test for bilateral degree of signal change. To investigate the effect of esotropia on different frequency bands, we performed an RM-ANOVA, during which the group was set as a between-subject factor and the frequency band was set as a within-subject factor. To achieve that, we designed an F-contrast (flexible factorial design, 2*5) for group*frequency band interaction. Significant clusters were extracted as regions of interest (ROIs). To explore differences in diseases and frequency bands further, post hoc, two-sample *t*-tests were performed with the averaged ALFF values extracted from these ROIs. Age, gender, and mean functional deficits^21^ were included as covariates. All significant tests were corrected for multiple comparisons with the Gaussian random field (GRF) theory correction at voxel level *P* < 0.005 and cluster level *P* < 0.05. Significant ROIs were reported in MNI coordinates of peak voxels.

#### Secondary Analyses

We also examined the relationship between ALFF values and clinical indications. ALFF values were extracted from ROIs showing significant differences in each frequency band and Spearman's correlation was calculated for each of the frequency bands. Multivariate linear regression analyses were performed subsequently. Clinical features included onset age of strabismus; duration of AACE; daily near work hours and eso-deviation at near and far. Statistical data were set to a significant level of *P* < 0.05.

## Results

### Clinical Characteristics

The mean age of patients with AACE is 26.44 ± 7.26 years old, including 12 male patients and 13 female patients, and the mean age of healthy controls is 26.32 ± 3.09 years old, including 12 male controls and 13 female controls. The clinical features are summarized in Table 1. There was no significant difference between groups in terms of gender, age, refractive error, or BCVA, except that the patients with AACE had uncorrected near work time that was longer than the control group (*P* < 0.05).

### The ALFF Alterations

**Figure 1.**
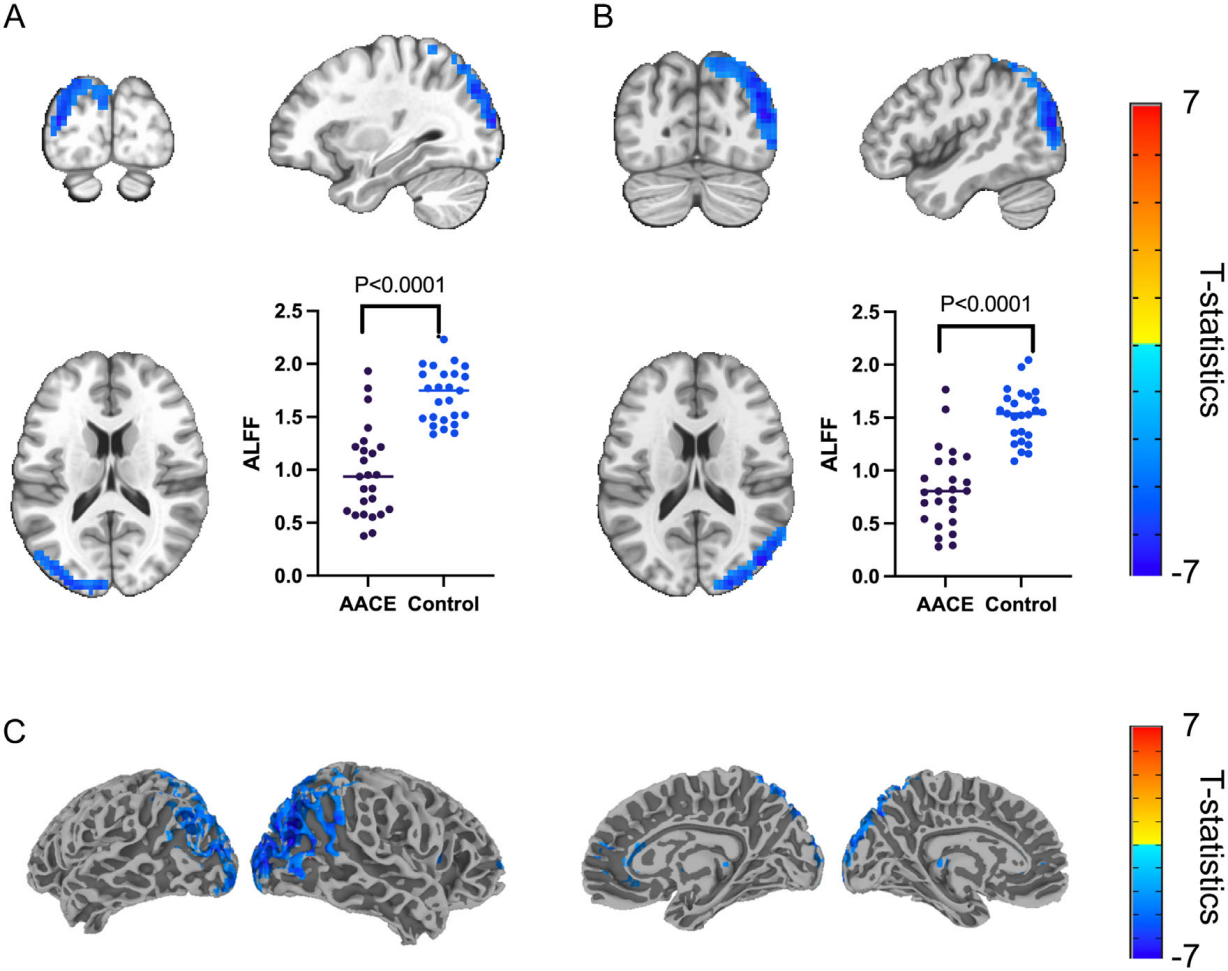
Signal decreased cortical regions in AACE versus healthy control group (*n* = 25, cluster *P* < 0.001). (**A**) Left brain in three axials, and the average ALFF signals from the whole region in patients with AACE and healthy controls. (**B**) Right brain in three axials, and the average ALFF signals from the whole region in patients with AACE and healthy controls. (**C**) Cortical surface projections of regions with signal decreased.

We first examined group differences of ALFF in the typical frequency band (0.01–0.08 Hz) and found a decreased signal in the primary visual cortex and dorsal pathway of the visual cortex bilaterally in patients with AACE. The results are listed in Table 2. The peak t was located in the mid occipital gyrus (MOG) on both cortical sides. We presented bilateral area from three axials respectively and also projected the differential area to the cortical surface, all of which are presented in Figure 1. The results indicated strongly bilateral symmetry (with a peak t of 7 in the left and 7.7 in the right).

**Table 2.** Signal Decrease Cortical Regions of AACE Versus Healthy Controls

Peak Information				Cortical Region	
	Peak t	[x, y, z]	Cluster Volume	Functional Area	Volume
Left	−2.95	[−31.5, −84, 28]	616	—	—
Left				Middle occipital gyrus	159
				Superior parietal gyrus	85
				Superior occipital gyrus	80
				Precuneus gyrus	70
				Cuneus	62
				Angular gyrus	31
Right	−2.96	[14, −59.5, 63]	915	—	—
Right				Middle occipital gyrus	207
				Superior parietal gyrus	116
				Superior occipital gyrus	112
				Angular gyrus	104
				Middle temporal gyrus	65
				Cuneus	46
				Precuneus	43
				Angular gyrus	35

**Figure 2.**
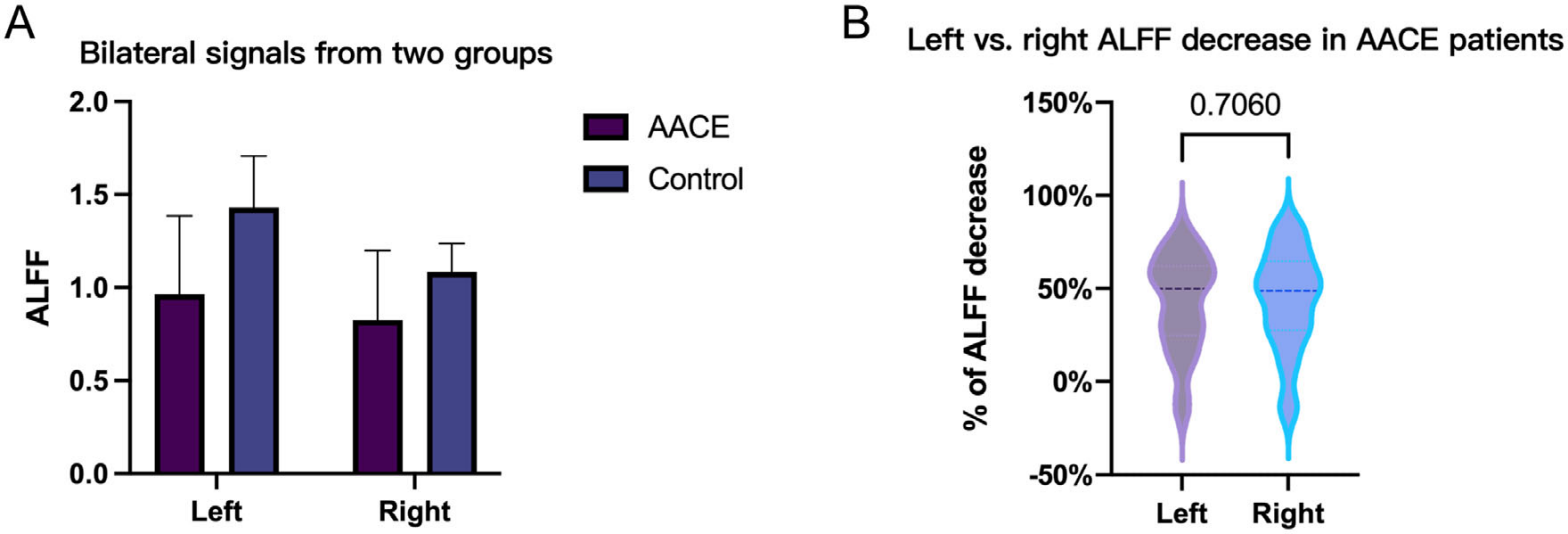
Difference of bilateral ALFF signal change (*n* = 25, cluster *P* < 0.001). (**A**) Left versus right AACE signals in two groups. (**B**) Percentage of signal decrease bilaterally in patients with AACE.

RM-ANOVA was performed then to further validate whether the ALFF difference was related to the disease. As was shown in Table 3, it was the group factor rather than the bilateral factor that resulted in the signal difference of the AACE group and the control group (*P* < 0.001). In addition, the decreased percentage of both sides also validated the bilateral symmetry of signal decrease in patients with AACE (Fig. 2).

**Table 3.** RM-ANOVA of Bilateral Signal in Classical Band in Two Groups

	SS	DF	MS	F (DFn, DFd)	*P* Value
Interaction	0.01	1	0.01	F(1, 96) = 0.11	*P* = 0.74
Left/right	0.65	1	0.65	F (1, 96) = 5.86	*P* = 0.02
AACE/control	12.86	1	12.86	F (1, 96) = 116.0	*P* < 0.001

**Figure 3.**
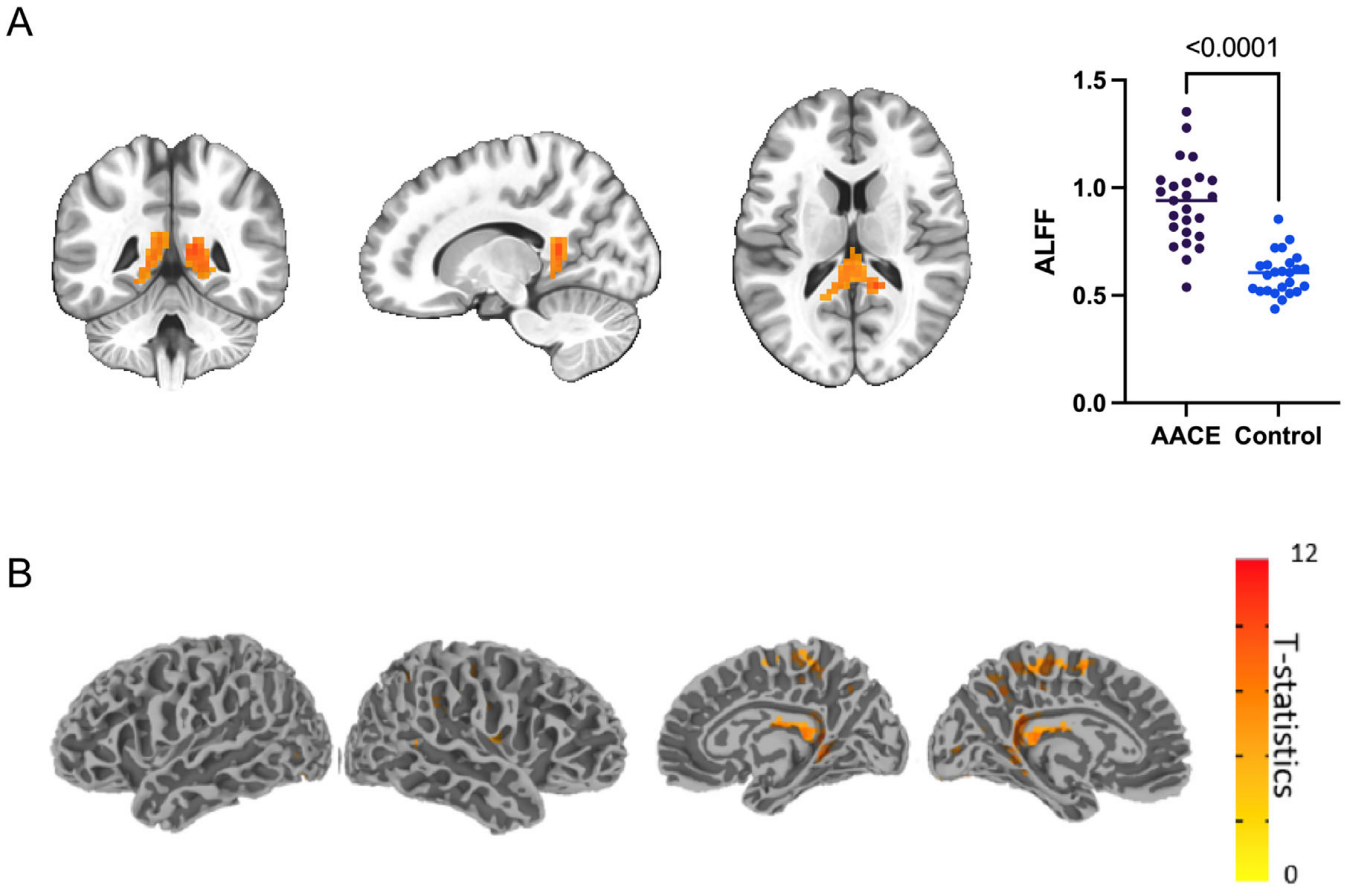
Signal increased cortical regions of AACE versus healthy controls (*n* = 25, cluster *P* < 0.05). (**A**) Signal increased cortical regions in three axials and the average signals extracted from two groups. (**B**) Signal increased cortical regions projected to cortical surface.

On the other hand, we also found signal increase in areas, especially bilateral precuneus areas and left fusiform gyrus (Table 4). The average signal in the mass area showed a significant difference, as shown in  Figure 3.

**Table 4.** Signal Increase Cortical Regions of AACE Versus Healthy Controls

	Peak Information			Cortical Region	
	Peak t	[x, y, z]	Cluster Volume	Functional Area	Volume
Right	5.99	14, −45.5, 14	1169	—	—
Right				Precuneus Gyrus	63
				Paracentral lobule	42
				Precentral gyrus	34
Left				Precuneus gyrus	94
				Fusiform gyrus	76
				Paracentral lobule	46
				Supplementary motor area	33

**Figure 4.**
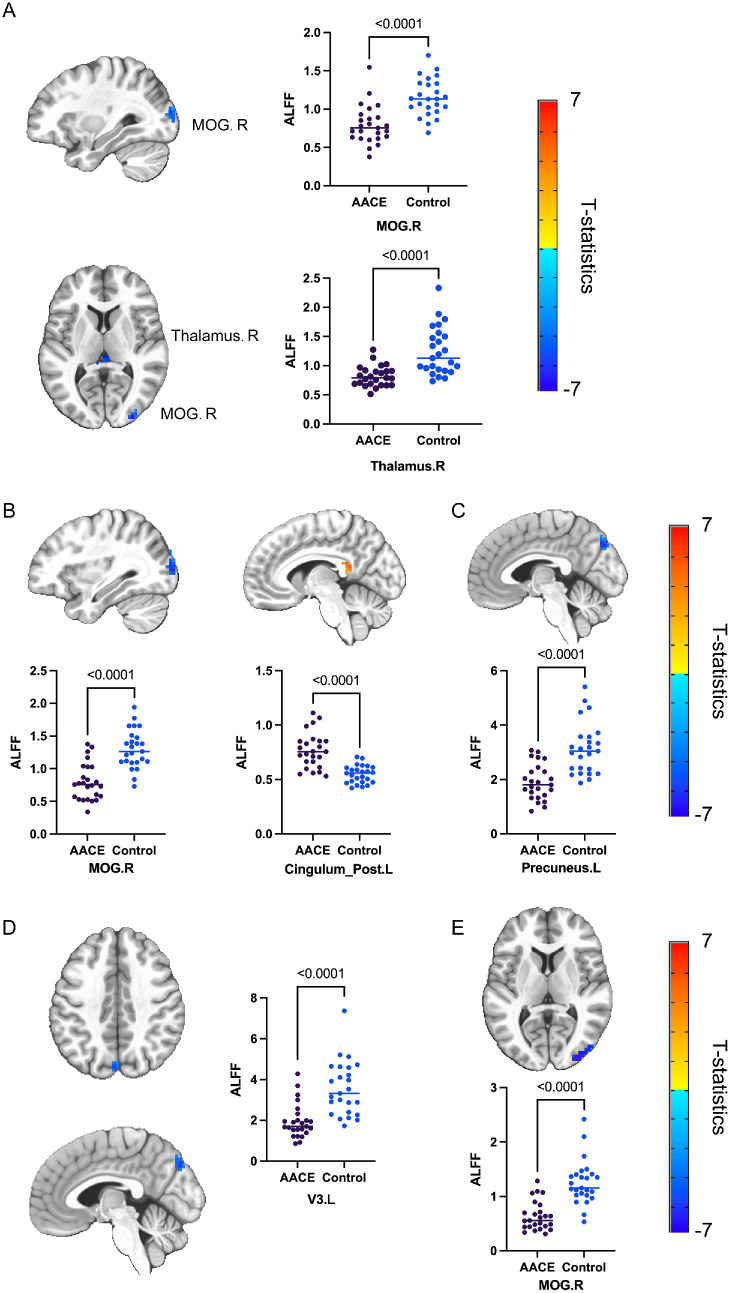
Significantly different areas between AACE and control groups in slow-2 to slow-6 frequency bands (*n* = 25, GRF-corrected cluster *P* < 0.05). Average signals extracted from subjects of two groups were shown in scatter chart. (**A**) Significantly different areas between groups in slow-2 band. (**B**) Significantly different areas between groups in slow-3 band. (**C**) Significantly different areas between groups in slow-4 band. (**D**) Significantly different areas between groups in slow-5 band. (**E**) Significantly different areas between groups in slow-6 band.

As denoted in the previous study, the frequency band was an important factor that may affect the signal. Therefore, we performed RM-ANOVA, and then, set the frequency band (5 grades from slow-2 to slow-6) as the within-subject factor and group as the between-subject factor. As is shown in Table 5, the results revealed the abnormal areas, including the thalamus, left precuneus gyrus, left temporal gyrus, left mid occipital gyrus, right mid occipital gyrus, and right angular gyrus. After a post hoc analysis for each sub-band, we presented regions with significant signal change in each sub-band of patients with AACE, respectively, in Table 5, including the decreased signal from patients with AACE in the bilateral thalamus and right V4 area of the slow-2 band, the decreased signal from patients with AACE in the right mid occipital area of the slow-3 band, the increased signal in the left posterior cingulate of the slow-3 band, the decreased signal in the left precuneus gyrus of the slow-4 band, the decreased signal in the left V3 area of the slow-5 band, and the decreased signal in the right mid occipital gyrus of the slow-6 band. The average signal of the whole mass was extracted respectively for each sub-band and is shown in Figure 4.

**Table 5.** RM-ANOVA Results of Group Factor × Frequency Band Factor

			Peak Coordinate (mm)			
Side	Region	F Value	x	y	z	Volume (Voxel)
**Bilateral**	Thalamus	36.77	0	−24.5	14	96
**Left**	Precuneus	47.28	−3.5	−77	45.5	17
	Temporal	29.00	−38.5	24.5	−24.5	15
	Mid occipital gyrus	24.08	−31.5	−80.5	38.5	8
**Right**	Mid occipital gyrus (V4)	36.33	35.0	−91.0	10.5	44
	Angular gyrus	23.87	45.5	−70	42	6

### Correlation Analysis

**Figure 5.**
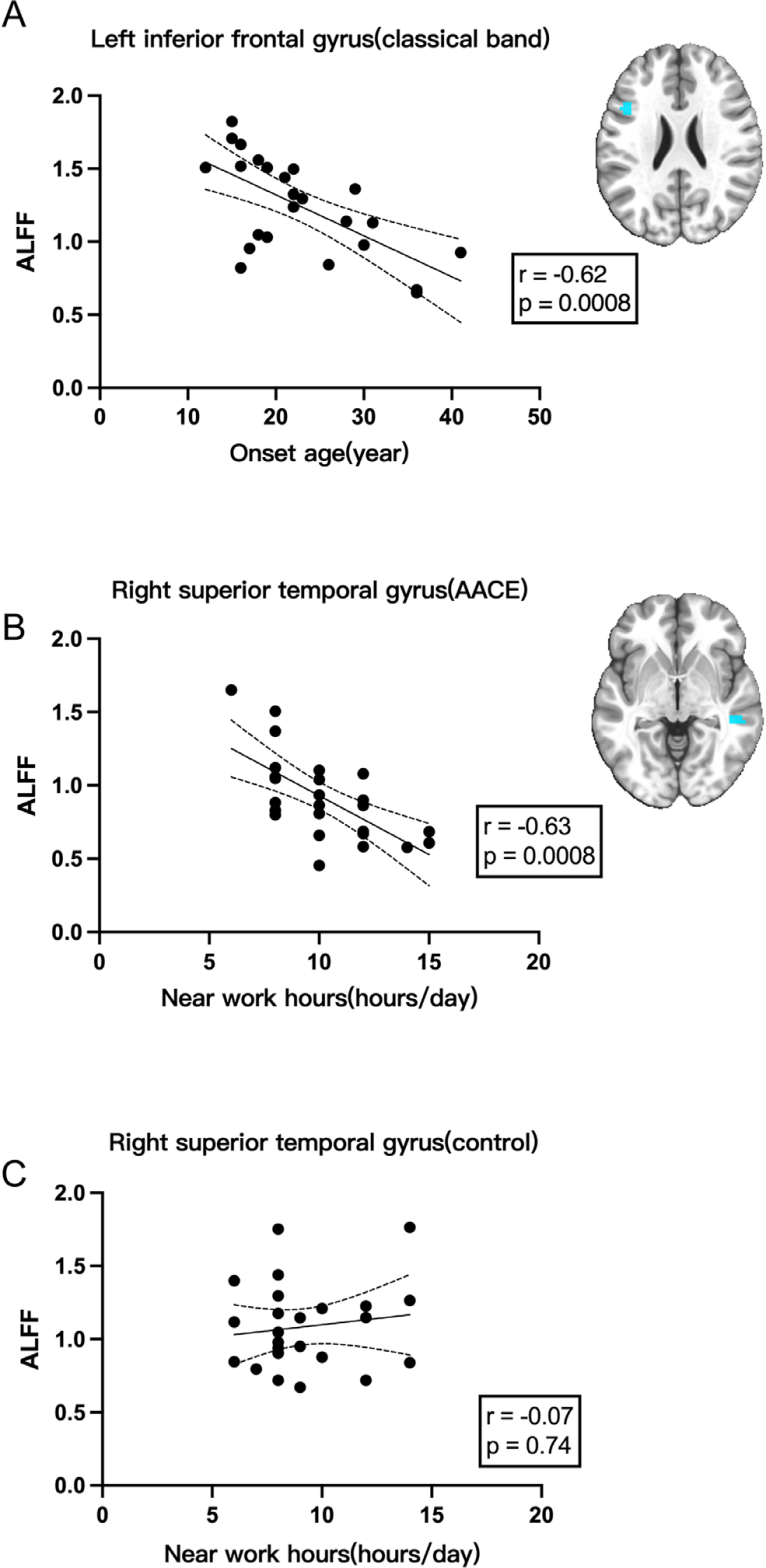
Negative correlations between average signals extracted form patients with AACE and clinical manifestations (*n* = 25, *P* < 0.05). (**A**) Average ALFF signals extracted from the left inferior frontal gyrus were negatively correlated with the onset age of disease. (**B**) In the AACE group, average ALFF signals extracted from the right superior temporal gyrus were negatively correlated with daily near work hours. (**C**) In the control group, average ALFF signals extracted from the right superior temporal gyrus had no significant correlation with daily near work hours.

**Figure 6.**
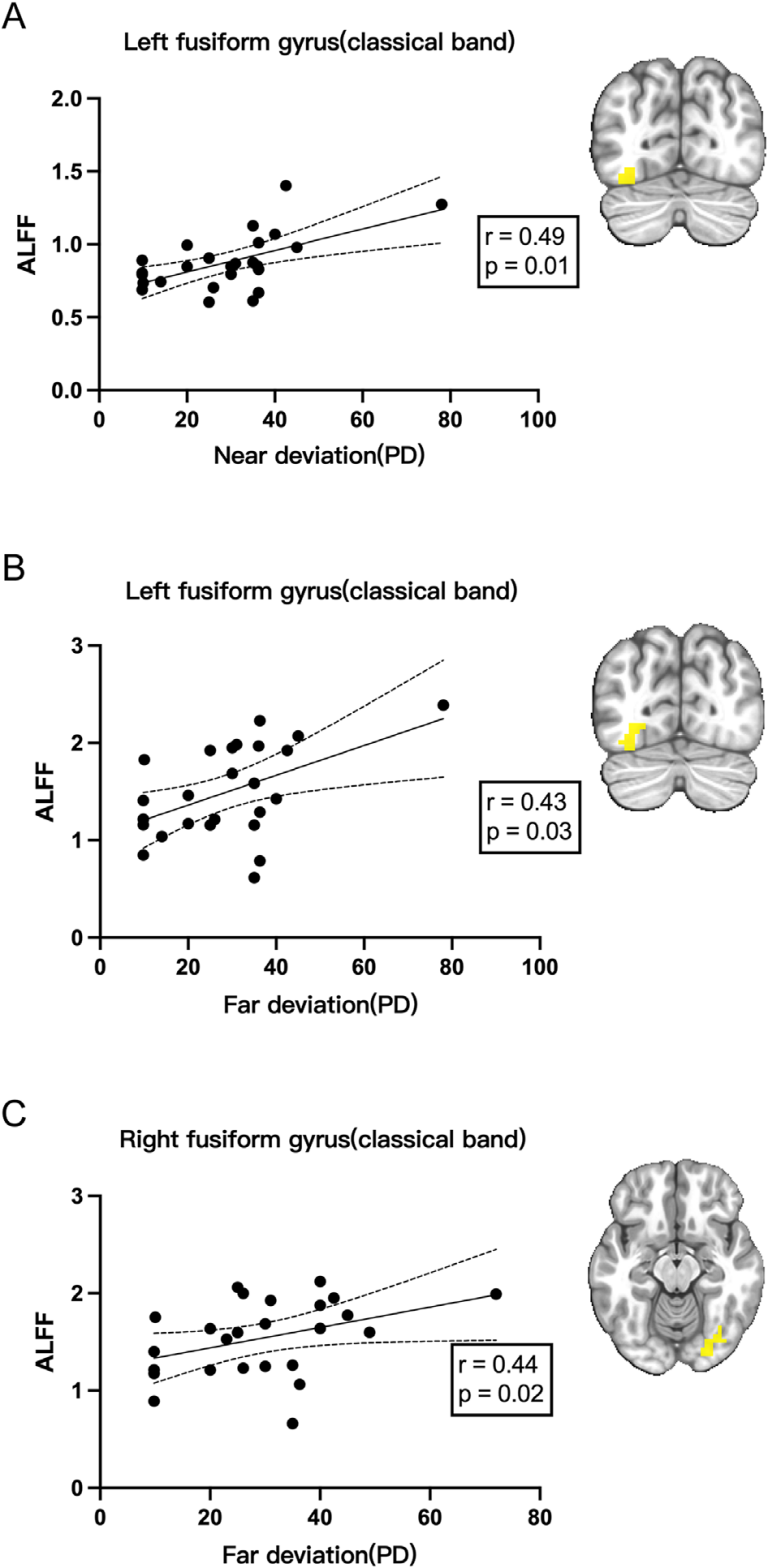
Positive correlations between average signals extracted form patients with AACE and clinical manifestations (*n* = 25, *P* < 0.05). (**A**) Average ALFF signals extracted from the left fusiform gyrus were positively correlated with the near deviation (PD). (**B**) Average ALFF signals extracted from the left fusiform gyrus were positively related with the far deviation (PD). (**C**) Average ALFF signals extracted from the right fusiform gyrus were positively correlated with the far deviation (PD).

To check if there was any correlation between abnormal ALFF signals and clinical features, correlation analysis was performed using the Spearman method. First, we defined those areas where patients with AACE ALFF signals of the classical frequency band were significantly different from those of the control group (including increase and decrease). Then, the average ALFF signals in those areas were extracted, which was used to perform correlation analysis with disease onset age, disease duration, daily near work hours, and deviation at far and at near, respectively. Finally, we found that the ALFF signals from the left inferior frontal gyrus was negatively correlated with the onset age of disease, and the signal from the right superior temporal gyrus was negatively correlated with daily near work hours, with results shown in Figure 5. At the same time, signals from bilateral fusiform gyrus were positively correlated with deviation, both at far and at near, with correlation results shown in Figure 6.

To further investigate the association of near work hours and AACE occurrence, we also did a correlation analysis for ALFF signals from the same position with the AACE group and the near work hours in the control group. As is shown in Figure 5C, in the control group, average ALFF signals extracted from the right superior temporal gyrus had no significant correlation with daily near work hours.

### Multivariable Linear Regression

We extracted the average ALFF signals from the abnormal areas of slow-2, slow-3, slow-4, slow-5, and slow-6 as well as the classical signal band, respectively. Then, we defined these signals as dependent variables and defined clinical manifestations, including onset age, disease duration, daily near work hours, and deviation (use deviation at far only in case of collinearity), as independent variables. After linear regression analysis, we found that in the classical band, onset age was the independent factor of signals from the left inferior frontal gyrus, and daily near work hours was an independent factor of signals from the right superior temporal gyrus, all of which are shown in Table 6.

**Table 6.** Multivariable Linear Regression Results of ALFF Signals in Different Bands and Clinical Manifestations

	Left Inferior Frontal Gyrus			Right Superior Temporal Gyrus		
	OR	95% CI	*P* Value	OR	95% CI	*P* Value
**Onset age**	−0.030	−0.045 to 0.014	0.0008^*^	0.008	0.006 to 0.022	0.26
**Disease duration**	−0.017	−0.051 to 0.017	0.32	0.001	−0.03 to 0.03	0.93
**Near work hours**	−0.031	−0.082 to 0.020	0.21	−0.079	−0.12 to 0.03	0.002^*^
**Far deviation**	0.002	−0.006 to 0.010	0.63	−0.002	−0.010 to 0.005	0.54

* *p* ≤ 0.05.

## Discussion

Although some studies have found the abnormal brain activities in resting state in strabismus,^11^^–^^14^ there are no previous studies that explore the brain activities of patients with AACE, to the best of our knowledge. In this study, ALFF signals were found to decrease in bilateral dorsal cortex and increase in bilateral precuneus areas and left fusiform gyrus. At the same time, the ALFF signal from the left inferior frontal gyrus was negatively correlated with the onset age of disease, and the signal from the right superior temporal gyrus was negatively correlated with daily near work hours, and signal from bilateral fusiform gyrus was positively correlated with deviation.

According to the Burian's classification of AACE,^22^ no patient enrolled in our study complained of occlusion history or monocular vision damage, thus they were excluded from the Swan type. Most of the patients could be classified into Bielschowsky's type^23^ because they all have myopia, except only one patient had slight hyperopia (less than +1.00 D binocularly) and was classified as Franceschetti type. It was noteworthy that although most of them had their myopia within -5.00 D, there were still 6 patients who were more severe than that. Previously, there were also researchers suggesting that Bielschowsky's AACE should include higher degree of myopia.^24^

Through analysis of resting-state ALFF signals, we found that patients with AACE had signals change in the superior occipital gyrus, superior parietal gyrus, angular gyrus, and precuneus area, which were mainly located in the dorsal regions of the visual pathway. At the same time, the cuneiform and middle occipital gyrus, which were located in the primary visual cortex, also showed decreased spontaneous signal. Because previous studies have revealed that in infantile esotropia the spontaneous signals from the posterior parietal cortex and angular gyrus had been enhanced after stimuli promoting fusion, so we speculate that after acute onset of esotropia, disruption of fusion reduced binocular driven signal, thus reducing the binocular information input to the advanced visual cortex, and the spontaneous signals from the advanced visual cortex would be decreased then. This inference can be verified by the spontaneous signal decrease of the classical frequency band from the primary visual cortex located in the cuneiform. Furthermore, previous studies have denoted the difference of information sub-bands conveyed, for example, low frequency band slow-6 may be associated with sense of pain and motor control deficits,^25^^,^^26^ whereas high frequency bands, slow-2, and slow-3 may contain some information, like spatial delineation and functional integration of brain regions.^27^^,^^28^ Slow-4 and slow-5 are more often found to have association with binocular vision.^29^^–^^31^ In this study, we found the left middle occipital gyrus/V3 and left precuneus region exhibited band-specific changes in the slow-4 and slow-5 sub-bands. The V3/middle occipital gyrus is located in the anterior segment of the visual pathway,^32^ and the precuneus region, as part of the superior parietal lobule,^33^ is located in the posterior segment of the dorsal visual pathway, receiving signal input from the V6 cortex.^34^ As the top form of binocular vision, stereopsis has been thought to be produced by the posterior segment of the visual system, especially the lateral occipital complex (LOC) and the posterior parietal cortex (PPC). Spontaneous signal reduction in those areas may be associated with fusion impairment and decreased stereopsis after the onset of esotropia.

At the same time, the precuneus area was also involved in the cortical regions where spontaneous signal increased. Considering the relationship between the precuneus area and the fusional function compensation above, whether and how different sub-areas have different functions in binocular fusion in the precuneus area still need our further investigation. The complexity of spontaneous signals in the precuneus cortex tells us that the precuneus cortex is important in binocular fusion function. In addition, the paracentral lobules, precentral gyrus, and supplementary motor areas all belong to the motor cortex. How are the increased signals in these areas related to the visual system? Is it a compensatory change to the reduction of visual input or negative feedback to the visual system? Further studies such as functional connectivity (FC) analysis are needed in order to make it clearer.

In our study, the signals in the left inferior frontal gyrus were inversely correlated with the onset age of disease, but no signal abnormalities were related to the disease duration. Spierer^24^ and Ohtsuki^35^ have previously reported that there is no correlation between visual function recovery and the timing of surgery in esotropia. This conclusion can also be verified in domestic studies. These clues remind us that we should pay more attention to the age of disease onset in the pathogenesis of AACE. The inferior frontal gyrus was found to be a key brain region in emotional and cognitive control circuit,^36^ further experiments need to be done to check if age is an independent factor in the pathogenesis of AACE.

In addition, previous researchers have reported that long-term near work hours, especially using smartphones, were significantly positively correlated with the incidence of type III AACE.^16^^,^^37^^,^^38^ In our study, we found that signals from the right superior temporal gyrus were correlated with near work hours, whereas this correlation did not satisfy the control group. In addition, after multiple variable regression analysis, near work hour was also an independent factor among others. Such correlations have also been reported in some retrospective studies. Some scholars believe that long-term tension of the medial rectus muscle due to near works can disrupt fusion, thus inducing the AACE onset.^38^ Because the function of the superior temporal gyrus is mainly related to hearing, language, and social networks, and our study has not found other brain regions that were related to near work hours, so further research is needed to dig out the associations.

Through correlation analysis, we found that the fusiform gyrus was positively correlated with eye deviation. The most commonly mentioned function of the fusiform gyrus is that it is fusiform face area (FFA), but the fusiform gyrus is not limited to the function of face recognition. The fusiform area is also responsible for recognizing subcategories of objects, functioning further in the visual system, and processing more integrated visual signals. As for whether this abnormality has an association with the “top-down” regulations in the visual system, and are there any social factors mixed in, further investigations need to be done.

As a crucial role in mapping the intrinsic functional architecture of the brain during rest, rs-fMRI reveals the functional differences in patients with AACE during resting state compared to healthy controls, suggesting the presence of functional abnormalities in certain cortical areas in patients with AACE. However, due to its limitations, it cannot yet elucidate the relationship between these functional abnormalities and visual impairments in patients with AACE, particularly binocular vision impairments. Therefore, in the future, we will design more experiments based on diversified techniques like task-based fMRI and behavioral experiments to further investigate along this direction.

## Conclusions

Using the ALFF signal of rs-fMRI, we found functional deficits in the primary visual cortex and dorsal pathway in patients with AACE. There were functional changes in the fusiform gyrus, and the greater the deviation angle, the higher the changing level. These findings reveal the association of AACE and the visual center, giving us more clues about the treatment of AACE.
